# Tumor-derived exosomes in ovarian cancer – liquid biopsies for early detection and real-time monitoring of cancer progression

**DOI:** 10.18632/oncotarget.22191

**Published:** 2017-10-31

**Authors:** Shayna Sharma, Felipe Zuñiga, Gregory E. Rice, Lewis C. Perrin, John D. Hooper, Carlos Salomon

**Affiliations:** ^1^ Exosome Biology Laboratory, Centre for Clinical Diagnostics, UQ Centre for Clinical Research, Royal Brisbane and Women’s Hospital, Faculty of Medicine + Biomedical Sciences, The University of Queensland, Brisbane, Australia; ^2^ Department of Clinical Biochemistry and Immunology, Faculty of Pharmacy, University of Concepción, Concepción, Chile; ^3^ Department of Obstetrics and Gynecology, Ochsner Baptist Hospital, New Orleans, Louisiana, USA; ^4^ Mater Health Services, South Brisbane, Australia; ^5^ Mater Research Institute, University of Queensland, Translational Research Institute, Woolloongabba, Australia; ^6^ Mater Ovarian Cancer Research Collaborative, Mater Adult Hospital, South Brisbane, Australia

**Keywords:** ovarian cancer, exosomes, biomarkers, early detection

## Abstract

Ovarian cancer usually has a poor prognosis because it predominantly presents as high stage disease. New approaches are required to develop more effective early detection strategies and real-time treatment response monitoring. Nano-sized extracellular vesicles (EVs, including exosomes) may provide an approach to enrich tumor biomarker detection and address this clinical need. Exosomes are membranous extracellular vesicles of approximately 100 nm in diameter that have potential to be used as biomarkers and therapeutic delivery tools for ovarian cancer. Exosomal content (proteins and miRNA) is often parent cell specific thus providing an insight or “fingerprint” of the intracellular environment. Furthermore, exosomes can aid cell-cell communication and have the ability to modify target cells by transferring their content. Additionally, via the capacity to evade the immune system and remain stable over long periods in circulation, exosomes have potential as natural drug agents. This review examines the potential role of exosomes in diagnosis, drug delivery and real-time monitoring in ovarian cancer.

## INTRODUCTION

Ovarian cancer is the most lethal gynaecological cancer surpassing other malignancies such as uterine and endometrial cancer [1]. The 5-year survival rate for late stage disease is approximately 20% (Australian Institute of Health and Welfare, 2014) whereas at an early stage, the survival rate is approximately 90% [2]. Additionally, relapse is almost inevitable in several cases and thus, there is a need for novel therapeutics and tools for both early diagnosis and identification of women at risk of developing ovarian cancer, as well as biomarkers for real-time monitoring of response to therapy [3]. Furthermore, ovarian cancer is a heterogeneous disease with differences between patients presenting at the molecular level [4, 5]. These differences often mean that whilst one treatment may be effective for a particular patient, it may have no effect in controlling disease for another patient. Therefore, targeted therapies and personalised medicine have become appealing in this field [5]. However, implementing targeted therapies is an arduous task and thus understanding disease progression from the primary tumor leading to metastasis is vital [6]. A primary factor underlying disease progression is cell-cell communication within the tumor microenvironment. This is a poorly understood process although extracellular vesicles, specifically exosomes, have received significant attention, being recognized as key mediators [7, 8].

Exosomes are small (∼100nm), membranous vesicles of endocytic origin and can be found circulating in biological fluids such as blood, urine and milk [9, 10]. Perhaps what makes them most interesting is the idea that exosomes can encapsulate information from the releasing cells, carry signals and relocate packages of information in the tumor microenvironment to ultimately aid metastasis [11, 12]. Moreover, it has been shown that the concentration of plasma exosomal proteins is positively correlated with ovarian cancer disease severity and/or progression [13].

Therefore, this review will summarise the recent advances that have been made in understanding the relationship between ovarian cancer and exosomes. Specifically, the role of exosomes in screening and early detection, as biomarkers for prognosis and drug resistance and as drug delivery vehicles will be discussed.

## OVARIAN CANCER

Despite current advances in research, ovarian cancer retains its position as the leading cause of gynaecological related deaths in females worldwide [1, 14]. Subtypes of ovarian cancer can be histologically differentiated based on tumor biological behaviour and risk factors [15]. The majority of ovarian tumors can be grouped into three major categories depending on the cell of origin, which can be either epithelial, germ or stromal cells although malignant ovarian tumors of epithelial origin are the most common [16]. Carcinomas account for approximately 90% of the cases of ovarian cancer and these can be further distinguished based on histopathology, molecular genetic analysis and immunohistochemistry in to five recognised subtypes. These subtypes are: serous (SC), clear-cell (CCC), endometrioid (EC), mucinous (MC) and transitional cell (Brenner) carcinoma [17, 18].

The International Federation of Gynaecology and Obstetrics (FIGO) [19] has divided the diagnosis of ovarian cancer in to four clinical stages including: Stage I, where the disease is only present in the ovaries; Stage II, where the disease has spread to the fallopian tube, uterus or below the pelvic brim; Stage III, where the disease affects outside the pelvis and either the abdomen or the lymph nodes or both areas; and Stage IV where the disease metastasises to distant areas such as the liver and/or spleen and outside the abdomen such as the lungs often resulting in pleural effusions. The high rate of ovarian cancer mortality can be attributed to the fact that often symptoms are dismissed resulting in an inability to detect the disease at an early stage. The nature of symptoms is generally vague with commonly reported factors being abdominal pain and/or swelling and changes in bowel habits [20]. Furthermore, methods for estimation of susceptibility are also lacking as there are no known genetic mutations that can be identified in all patients presenting with a particular subtype of ovarian cancer.

### Estimation of susceptibility

Nonetheless, the most well-known factors involved in ovarian cancer susceptibility are mutations in the BRCA1 and BRAC2 genes [21] which are also related to breast cancer susceptibility. The involvement of these genes was identified early on when it was shown that BRCA1 mutations resulted in increased lifetime risk of developing breast-ovarian cancer with multiple cases reported in families [22]. Later research suggested that genetic testing for mutations in BRCA1 and BRCA2 be considered where there is a family history of either breast or ovarian cancer. However, genetic testing to determine susceptibility is recommended in cases with either Jewish ancestry or a family history of breast and/or ovarian cancer [23]. Another disadvantage to genetic testing is that it is expensive and therefore should be aimed at individuals most at risk. To identify these individuals, several mathematical models have been proposed and the advantages and disadvantages of these models have been discussed in Antoniou et al (2004) who have also proposed an improvised method [24].

Due to the limitations presented by genetic testing, literature has shifted towards different techniques for susceptibility estimation such as, studying smaller molecules, example, RNA and their contribution to disease. In 2016, Permuth and colleagues suggested that RNA editing, which is a post-transcriptional modification where adenosine is converted to inosine by a family of enzymes known as adenosine deaminases acting on RNA (ADAR), may have a role in epithelial ovarian cancers. ADAR expression levels have been found to be significantly greater in peritoneal fluid obtained from patients with epithelial ovarian cancer compared to patients with benign disease [25]. They concluded that examining ADAR genotyping and/or expression level could be a potential biomarker or risk factor [25]. It has also recently been shown that certain genetic variants lead to increased susceptibility to all subtypes of ovarian cancer whereas other variations confer subtype-specific susceptibility [26].

Approximately 20% of patients with high-grade serous subtype disease carry mutations (both somatic and germ-line) in the BRCA1 and BRCA2 genes [27]. However, patients with germ-line mutations have better overall survival rates. Mutations in other genes and genetic pathways such as KRAS, BRAF, TP53, PIK3CA, AR1D1A and HER2 have also been associated with the other subtypes of ovarian cancer as well as specific germ-line single nucleotide polymorphisms (SNPs) [26]. In addition to genetic factors, environmental factors such as obesity and smoking have been shown to have a correlation with increased risk [28]. An inverse correlation between age of menarche and risk of disease was also noted [29]. The identification of genetic mutations provides an estimation of susceptibility, however remains an expensive option.

### Metastasis and negative survival rates

Another factor that is negatively correlated with survival is the fact that ovarian cancer is a heterogeneous disease and therefore the *“one size fits all”* approach cannot always be applied to treatments. The different subtypes of ovarian cancer have diverse aetiologies and different sensitivity to treatments [26]. Current treatments most commonly include surgical tumor de-bulking and chemotherapy [30]. The chemotherapeutics generally used are carboplatin and paclitaxel, however there is often relapse [30]. The progression between the different stages of ovarian cancer is minimally understood although cell signaling is thought to be involved. Nonetheless, there are three known pathways through which epithelial ovarian cancer can metastasise: transcoelomic, hematogenous and lymphatic spread [31, 32]. Of these, the most common route of metastasis is via transcoelomic dissemination although this process is poorly understood as it differs from the traditional model of cancer metastasis, the hematogenous spread of cancer cells. [33].

Invasion of the tumor cells into the pelvic and abdominal peritoneum is often representative of tumor aggressiveness [34]. This invasion of disease into a body cavity is defined as transcoelomic metastasis. A detailed review of this process is provided by Tan and colleagues [31]. Briefly, the tumor can arise from three potential sites: the ovarian surface, mesothelium lined peritoneal cavity or the fallopian tube [35]. Following progress in carcinoma tumorigenesis, the cells undergo the Epithelial to Mesenchymal transition (EMT) to become more motile. Single tumor cells or tumor cell clusters then disseminate from the primary tumor into the peritoneal cavity and attach to abdominal organs [34]. Upon attachment, the tumor cells return to their epithelial phenotype and proliferate rapidly leading to increased disease burden. Metastasis and aggressive cell division are often indicators of late stage disease with poor prognosis. Therefore, it is essential that the tumor can be detected when it is limited to the ovaries to improve patient outcomes. However, early detection of ovarian cancer remains a challenge as current detection techniques fall short leading to increased mortality rates.

### Current detection techniques

Cancer Antigen-125 (CA-125) and Trans-vaginal imaging are currently routinely used as screening tests for ovarian cancer [36]. CA-125 is a membrane bound glycoprotein with a high molecular weight [37] and a few splice variants which share the same trans-membrane and intracellular regions [37]. It has been linked to the Epithelial Growth Factor Receptor (EGFR) pathway and is often found on the surface of cells that differentiate to form Mullerian-type epithelium (serous, clear cell and endometrioid ovarian carcinomas) [38]. CA-125 can also be released into the circulation and thus detected using antibodies which can be characterised as OV197, M11 or OC125 type antibodies [39]. Trans-vaginal ultrasound is useful for imaging the ovaries and surrounding organs, however, it is often difficult to distinguish tumor formation from functional cysts in pre-menopausal ovaries. CA-125, on the other hand, is not useful in early diagnosis as up to 50% of Stage I ovarian cancer patients do not express elevated levels [38]. CA-125 levels can also lead to false positive results with raised levels seen in several other gynaecological conditions such as ovulation, menstruation and pregnancy as well as cancers such as fibroids and cancer of the bladder and liver [36]. A randomised controlled trial also reported that simultaneous screening with CA-125 levels and Transvaginal ultrasounds did not reduce mortality when compared to standard care [40]. Since circulating CA-125 concentration has low sensitivity and specificity, other avenues, such as exosomal CA-125 have also been explored.

Zhao et al (2016) reported the development of a microfluidic chip approach to isolate exosomes that expressed certain markers (Epithelial Cell Adhesion Molecule (EpCAM), CA-125 and CD24) [41]. It was also noted that when anti-EpCAM or anti-CA-125 antibodies were used to capture the exosomes, there was a greater number of exosomes captured in patient samples compared to controls [41]. Therefore, although circulating CA-125 may have low sensitivity and specificity, exosomal CA-125 may be used to distinguish between healthy and diseased patients. However, the use of CA-125 positive exosomes as potential biomarkers for ovarian cancer in a clinical setting is yet to be validated. Overall, due to the lack of early detection tools, protective salpingo-oophorectomy, which is the removal of the ovaries in addition to the fallopian tubes, is recommended [42]. However, this is not always feasible as patients often wish to preserve fertility.

It is thus clear that improved screening tests are required for early detection of disease. These screening tests need to fit a specific criteria including: the test can be performed easily, is inexpensive, clinically validated and highly sensitive and specific [36, 43]. Several approaches that fit these criteria have been proposed to combat the shortcomings of the current techniques, including the use of other tumor markers such as CA-15-3 and carcinoembryonic antigen (CEA) which are both elevated in patients with ovarian cancer [44]. However, these markers have poor correlation with the clinical course of disease.

### Circulating molecules in early detection

Smaller molecules such as circulating miRNA and cell-free DNA (cf-DNA) have also received attention in recent literature although progress is limited. Kamat and colleagues quantified cf-DNA and reported that patients with epithelial ovarian cancer had higher levels compared to healthy individuals [45]. Furthermore, increased cf-DNA levels were correlated with reduced survival rates. However, sensitivity of cf-DNA for detecting early stage disease was only 55%. Shao and colleagues found that patients with ovarian cancer had increased levels of cf-DNA and that it also increased with advanced stage disease with sensitivity at 88.9% [46]. Although the use of cf-DNA concentration is an appealing idea, it is unreasonable as cf-DNA concentration is often increased in tumors affecting multiple organs.

This is also a limitation in the use of miRNA expression as an ovarian cancer early detection tool. Previous studies have identified changes in the expression of multiple miRNA between patients with epithelial ovarian cancer and healthy controls such as Let-7 and miR-205. Let-7 levels decreased in patients with ovarian cancer whereas miR-205 levels were higher [47]. However, increased expression of miR-205 is also seen in other types of cancers such as endometrial carcinomas and squamous cell lung carcinoma. Other pitfalls associated with the use of circulating miRNA as a biomarker involve the unavailability of suitable animal models for miRNA research, lack of reproducibility and the costs associated with miRNA profiling [48, 49]. An in-depth discussion of these challenges can be found in Witwer (2015) and Tiberio et al (2015) [49, 50]. In response to these issues, the focus has shifted towards novel techniques for examining miRNA and protein. Exosomes, which encapsulate and hence protect from degradation multiple molecules such as miRNA, have gained increasing popularity in literature. They provide a minimally invasive method to gain an insight into the tumor microenvironment.

### EXOSOMES MAY IMPROVE DIAGNOSTIC PREDICTIVE VALUE

Cell-cell signaling as well as particles released by tumor cells to aid cellular communication remains a major focus. However, cells release a diverse population of factors including signalling molecules, transmitters, apoptotic bodies and microvesicles. These factors combined with the multitude of other abundant proteins (65-97% of total proteins) in the blood render it difficult to identify molecules of interest from tumor cells as there is a high signal-to-noise ratio [51, 52]. To tackle this issue, focus has shifted towards extracellular vesicles, specifically exosomes, which provide a better representation of the cellular environment compared to other free molecules. They have been highlighted as it has previously been shown that tumor cells (compared to normal cells) release a greater concentration of exosomes [53, 54]. Further, exosomes have been implicated in aiding pre-metastatic niche formation thus preparing secondary sites for metastasis [6, 7, 55, 56]. Therefore, exosomes (highly stable membranous vesicles) may increase diagnostic accuracy by decreasing the signal-to-noise ratio [51]. Furthermore, exosomes are released by all cells that have been examined till date, including both normal and tumor cells [57], and vary in size with a commonly accepted diameter of approximately 30-100nm [58, 59].

### Exosome biogenesis and release

The biogenesis of exosomes begins with an inward budding of the plasma membrane leading to the formation of an early endosome. The early endosome then matures to a late endosome. Invagination of the late endosomal membrane leads to the formation, within the endosome, of intraluminal vesicles known as multivesicular bodies. The multivesicular body can then either fuse with a lysosome leading to degradation or fuse with the plasma membrane of the cell leading to the release of exosomes [60-62]. Secretion of exosomes through the fusion of the multivesicular body requires Rab GTPases such as Rab27a and Rab27b [63]. However, release can be prevented using ceramide biosynthesis inhibitor, GW4869 [64]. Although exosome biogenesis has been extensively studied, mechanisms of communication with target cells are yet to be fully elucidated but are thought to be via one of three pathways: the cell receptor-exosome ligand interaction, exosome fusion with the target cell leading to release of exosomal cargo, and internalisation of exosomes by target cells through phagocytosis or lipid-raft mediated endocytosis [65-70].

Exosomes express a range of proteins including those related to multivesicular body biogenesis, such as Alix, TSG101 and members of the tetraspanin family [9]. The diverse origins of exosomes suggest roles in facilitating communication under both normal and pathological conditions. However, the pathway through which they undertake this role is yet to be understood. In addition to roles in cell-cell communication, exosomes are also being evaluated as potential biomarkers particularly because the contents of these extracellular vesicles are cell type specific [2, 71]. It is also thought that exosome content can be used to determine the metabolic state of the cell of origin.

### Exosomes in the tumor microenvironment

Exosomal cargo is composed of a range of molecules including but not limited to proteins (e.g. cytoskeletal proteins, membrane and fusion proteins and heat shock proteins (HSPs)), cell surface receptors and miRNAs [58]. Interestingly, the ability of exosomes to transport molecules such as proteins and miRNA also suggests roles in transforming healthy cells to cancerous cells resulting in a premetastatic niche [72]. MicroRNAs (miRNAs) are small non-coding RNAs that influence gene expression by targeting complementary messenger RNA (mRNA) [73]. miRNAs can aid tumor progression as oncogenes upregulated in disease and as tumor suppressors’ downregulated in disease.

The implications of exosomes in various biological processes as well as in pathological conditions have been examined although further research is required. For example, it has been reported that tumor-derived exosomes can promote oncogenic activity in recipient tumor cells. Moreover, exosomes can also aid in the development of suitable microenvironments for tumor growth and progression through processes such as angiogenesis. It has been shown that tumor-derived exosome can promote angiogenesis by activating myofibroblasts and endothelial cells [74]. Exosomes can assist microenvironment development by educating bone marrow-derived cells (BMDC) which are vital to tumor development [12]. BMDCs treated with exosomes isolated from highly metastatic melanoma cells resulted in an increased metastatic behaviour displayed by primary tumors through the education of bone marrow progenitors. The role of exosomes in transferring the oncoprotein MET, which is involved in metastasis by affecting mobilisation, was also elucidated [12]. Furthermore, exosomes can communicate with the immune system to counter anti-tumor responses thereby promoting pro-tumorigenic behaviour [56]. Furthermore, Philip et al (2015) showed that exosomes obtained from highly metastatic cells can transfer metastatic phenotypes through Epithelial to Mesenchymal Transition (EMT) related proteins to poorly metastatic cells [75].

Therefore, there is increasing research involving extracellular vesicles or exosomes, however, a major challenge is to understand the exact mechanisms underlying the role of exosomes in healthy cell transformation. Thus, the current focus is to decipher these mechanisms to facilitate early detection and prevention of ovarian cancer. Additionally, a challenge that remains in the field of extracellular vesicles is an ability to isolate and characterise exosomes as there is no consensus in current literature regarding an exosome isolation protocol [76, 77].

Table 1 presents the different techniques involved in exosome isolation. Due to a lack of characterisation methods, there is confusion in the nomenclature with several different types of microvesicles being referred to as exosomes. However, the implications of this challenge and different isolation techniques have been extensively discussed elsewhere [77-83]. Exosomes and their cargo (proteins, miRNA etc.) have been proposed as valuable resources for understanding the metabolic status of cells and can be utilised as prognostic and diagnostic biomarkers in the context of tumors. A summary of the studies involving extracellular vesicle content and ovarian cancer is presented in Table 2.

**Table 1:** Summary of studies involving isolation of EVs in ovarian cancer *(1999-2017)*

EVs	Sample Type(s)	Disease Type	EV Isolation Method	Biological Process/ Results	Reference
**Biological Fluids**					
Shed membrane vesicles	Ascitic Fluid Serum	Papillary adeno-carcinoma of the ovary	Centrifugation	Membrane vesicles in the plasma of patients are similar to membrane vesicles obtained from cell lines established from the same patient.	[103]
Membrane bound vesicles	Ascites	Stage I-IV malignant ovarian disease	Centrifugation	Vesicles from all malignant ascites stimulated invasion in cultured malignant ovarian epithelium.	[90]
Exosomes	Ascites	Malignant ovarian cancer	Centrifugation and Sucrose Gradient	Exosomes isolated from ascites have tumor specific antigens which can be recognised by DCs.	[104]
Exosomes	Serum	Serous papillary adeno-carcinoma (Stages I-IV), benign ovarian adenoma, NEOD	Modified magnetic activated cell sorting (MACS) procedure using EpCAM	miRNA profiling of exosomes can reflect the tissue miRNA profile.	[105]
Exosomes	Serum	High-grade serous ovarian cancer	Centrifugation	Exosomes from ovarian cancer patient plasma contain Claudin-4.	[86]
Exosomes	Ascites	Epithelial ovarian cancer	Centrifugation and Density Gradient	Exosomes exist in the ascites of 85.4% of ovarian cancer patients; however, they did not have any significant *in vitro* effect on tumor growth or apoptosis.	[106]
Exosomes	Serum	Epithelial ovarian cancer	Commercial Kit	Epithelial ovarian cancer derived exosomes can be up-taken by macrophages and these exosomes can induce differentiation of macrophages to a more tumor-associated macrophage like phenotype.	[107]
Exosomes	Ascites	Ovarian cancer	Nano-plasmonic (nPLEX) assay	Surface-plasmon resonance approach for detection of exosomal proteins. Ascitic samples were used as they contain a large quantity of exosomes. The exosomes can be isolated by elution from the device.	[108]
Exosomes	Plasma	Ovarian cancer	ExoSearch Chip (Immunomagnetic beads)	Three exosomal tumor markers (CA-125, CD24 and EpCAM) were used to isolate exosomes from ovarian cancer patient plasma.	[41]
Exosomes	Plasma	Ovarian cancer	Graphene oxide/polydopamine (GO/PDA) nanointerface chip	The GO/PDA coating increased the immuno-isolation efficacy whilst decreasing non-specific exosome adsorption.	[109]
**Cell-Conditioned Media**					
Exosomes	Ovarian cancer, colon cancer and breast cancer cell lines		Centrifugation and Sucrose Gradient	Claudins can be identified in exosomes isolated from CCM.	[86]
Exosomes	Ovarian cancer, embryonic kidney and neuroglioma cell lines		Centrifugation and Sucrose Density Fractionation	Ovarian cancer cells internalise exosomes via endocytic routes.	[7]
Exosomes	Ovarian cancer cell lines and ADSCs		Centrifugation and Discontinuous Sucrose Gradient	Treatment with ovarian cancer cell line derived exosomes led to ADSCs displaying tumor-associated myofibroblast characteristics.	[110]
Exosomes	Ovarian cancer cell lines		Centrifugation, Density Cushion and Sucrose Gradient	Proteomic analysis of OVCAR-3 and IGROV1 exosomes.	[87]
Exosomes	Ovarian cancer cell lines		Centrifugation and Sucrose Cushion	miRNA profiling of OVCAR-3 and SKOV-3 exosomes.	[53]
Exosomes	Epithelial ovarian cancer cell lines.		Centrifugation	Proteomic analysis of OVCAR-3, OVCAR-433, OVCAR-5 and SKOV-3 exosomes.	[111]
Exosomes	Ovarian cancer and human embryonic kidney cell lines		Centrifugation and Commercial Kit	HEK293 cells were able to uptake IGROV1 and OV420 exosomes. Treatment with IGROV1 cells led to increased invasion and migration of HEK293 cells.	[112]
Exosomes	Ovarian cancer cell lines and HUVECs		Centrifugation	Exosomes from cancer cell lines can enhance proliferation, migration and tube formation with CAOV-3 exosomes exerting a greater effect then SKOV-3 exosomes.	[113]
Exosomes	Late stage ovarian cancer cell lines		Centrifugation	Proteomic analysis showing that the pentose pathway is a major mechanism in exosomes mediated cellular communication.	[114]
Exosomes	Ovarian cancer cell lines, normal adipocytes and cancer associated adipocytes		Centrifugation	Exosomes can deliver miR-21 from stromal cells to tumor cells leading to chemo-resistance.	[92]
Exosomes	Human ADSCs		Commercial Kit	Exosomes from human ADSCs can restrict wound-repair, colony formation and proliferation of ovarian cancer cell lines (A2780 and SKOV-3).	[115]
Exosomes	Ovarian cancer cell lines		Centrifugation and Commercial Kit	Removal of miR-6126 through exosomes results in increased oncogenic behavior of cancer cells.	[116]
Exosomes	Ovarian cancer and ovarian epithelial cell lines		Centrifugation and Filtration	Exosomes transfer CD44 to surrounding mesothelial cells leading to disease progression.	[6]
Exosomes	Ovarian cancer cell lines		Centrifugation	Exosomes are able to transfer platinum resistance from resistant cells to their sensitive counterparts.	[117]
EVs	Ovarian cancer and mesothelial cell lines		Filtration and Centrifugation	EVs from aggressive cells induce metastatic characteristics in tumors. Cancer cell derived EVs also induce apoptosis in mesothelial cells both *in vitro* and *in vivo*.	[118]

EVs: Extracellular Vesicles, NEOD: No Evidence of Ovarian Disease, DCs: Dendritic Cells, miRNA: microRNA, EpCAM: Epithelial Cell Adhesion Molecule, CCM: Cell-Conditioned Media, ADSCs: Adipose Tissue Derived Mesenchymal Stem Cells, HUVECs: Human Umbilical Vein Endothelial Cells.

**Table 2:** EVs as potential diagnostic biomarkers of ovarian cancer *(2009-2017)*

EVs	Source(s)	Disease Type	Marker(s)	Results	Reference
Exosomes	Serum	Serous papillary ovarian adenocarcinoma, benign ovarian adenoma and no evidence of disease	miR-200c miR-214	Tumor exosomes had similar expression of certain miRNAs when compared to the tumor tissue. Expression of miR-200c and miR-214 was not significantly different when compared between stages but was significant when compared to benign disease.	[105]
Exosomes	Plasma	Ovarian cancer patients and healthy volunteers	Claudins (Claudin-4)	Claudins can be identified as part of exosomes shed by ovarian cancer cells in culture. Further, 32 out of 63 ovarian cancer patient plasma contained Claudin-4 positive vesicles compared to 1 out of 50 for healthy volunteers.	[86]
Micro-particles	Blood/ Plasma	Ovarian tumors with unknown histology	Concentration of particles	Concentration of micro-particles was not enough to distinguish between benign and malignant cases. However, patients with ovarian cancer had higher levels of activated platelet-derived micro-particles compared to benign disease patients.	[119]
Exosomes	Ascites	Ovarian cancer patients and portal alcoholic cirrhosis patients.	Proteins	40% of the proteins found in malignant ascites could also be found in exosomes. Exosomes derived from the malignant ascites had increased exosomal cargo.	[120]
Microvesicles	Plasma	Patients with an adnexal mass of unknown etiology	Microvesicle-associated Tissue Factor Procoagulant Activity (MV TF PCA)	Patients with ovarian cancer had an increase in the concentration of MV TF PCA	[121]
Exosomes	Plasma	Malignant and benign ovarian disease	Phosphatidylserine (PS)	PS-expressing exosomes could be used to distinguish patients with ovarian malignancies from patients with no disease.	[122]
Cell Lines					
Exosomes	OVCAR-3 and IGROV1 (ovarian cancer cell lines)		Proteins	Proteomic analysis of exosomes derived from the cell lines showed the presence of cell specific proteins implicating a role for exosomes as potential diagnostic biomarkers.	[87]
Exosomes	SKOV-3 and OVM (ovarian cancer cell lines)		LGALS3BP (sialglycoprotein)	LGALS3BP was identified as an Exosomal marker from SKOV-3 ovarian carcinoma cells and N-glycans from the exosomes were characterised.	[123]
Exosomes	OVCAR-3, OVCAR-433, OVCAR-5 and SKOV-3 (epithelial ovarian cancer cell lines)		Proteins	Proteomic analysis of exosomes showed the presence of epithelial ovarian cancer tissue proteins.	[111]
Extracellular vesicles (EVs)	OVMz (ovarian carcinoma cells)		Glycans	The presence of galectin-3-binding protein was noted in the EVs isolated from the OVMz cells and high expression levels of galectin-3-binding protein are associated with shorter survival.	[124]
Exosomes	OVCA429 and HO8910PM (ovarian carcinoma cell lines)		G6PD, transketolase and transaldolase 1	Exosomes from both the cell lines contained G6PD, transketolase and transaldolase 1 which are part of the pentose phosphate pathway and may be diagnostic of late stage cancer.	[114]

### Exosomes as early detection biomarkers

The proposal of personalised medicine by Kewal Jain in 1998 has drastically changed the approach towards cancer research and therapeutics [84]. However, the application of personalised medicine to a large population may only be possible if detailed patient information regarding tumor characteristics or the tumor signature is available. One method to achieve such information is to examine tumor-derived exosomes which are released from the endosomal compartments of a tumor cell, capturing vital information from within the cell. Advantages of using exosomes as biomarkers are that it is found within circulation, allowing for minimally invasive isolation, they contain specific molecules that can provide information about the parent cell as well as the probable target cells and that exosomes can protect information carrying molecules from degradation [84]. Garnier and colleagues (2013) proposed the use of exosomes as biomarkers as they noted that the exosomal content varied depending on whether the cell of origin had an epithelial or mesenchymal phenotype [85]. Taylor and Taylor (2008) compared the miRNA profile of ovarian tumor biopsies with the miRNA profile found within circulating EpCAM positive exosomes [13]. Interestingly, it was shown that the expression levels of specific miRNAs were similar between biopsies and exosomes from the same patient with correlations ranging up to 0.90 [13]. Furthermore, it was noted that the exosomal miRNA profile varied depending on disease state (benign or cancerous).

In 2009, Li et al showed that exosomes found in the circulation of approximately 32 (out of 63) patients with ovarian cancer contained claudins whereas only one out of 50 healthy individuals had claudin expressing exosomes present in their circulation [86]. Claudins are trans-membrane proteins which have been found to have increased expression in the context of ovarian cancer. Furthermore, claudin-3 and claudin-7 expression have an inverse correlation with survival in patients with ovarian cancer [86]. However, determining claudin expression requires a tumor biopsy which can often be an invasive procedure. Therefore, Li and colleagues examined whether exosomes contained claudins and if this could provide a less invasive diagnostic tool. Using claudin-4 positive exosomes as a biomarker, they obtained 51% sensitivity and 98% specificity [86]. Proteomic analysis by Liang et al showed that ovarian cancer cell line derived exosomes contained proteins that were cell specific thus providing avenues for new biomarkers [87].

Kobayashi et al (2014) showed that highly invasive ovarian cancer cell line, SKOV-3 derived exosomes had greater expression of the miRNA, Let-7, when compared to the poorly invasive cell line, OVCAR-3. Additionally, a correlation between the miRNA profiles of OVCAR-3 cell-derived exosomes and the OVCAR-3 parent cells was noted [53]. Vaksman et al (2014) profiled exosomal miRNA expression to analyse the potential of exosomal miRNA as prognostic tools [88]. They found that exosomal miRNAs-21, 23b and 29a correlated with poor survival and it was previously described that miR-21 and miR29a are overexpressed in the serum of ovarian cancer patients [89]. Overall, they stated that cancer cells may be packaging specific miRNA into exosomes which are then released into the effusion fluid. The exosomes can then educate the mesothelial cells leading to the diffusion of the tumor spheres throughout the peritoneal cavity [88].

Therefore, the potential of exosomes as diagnostic and prognostic biomarkers was hypothesised and it was found that several molecules native to the tumor cells/tissue were expressed in exosomes. This is promising as the expression of low abundance molecules may be masked in circulation but detected in exosomes. In 2004, Graves et al showed that matrix-metalloproteinases MMP-2 and MMP-9 which are related to ovarian cancer metastasis are concentrated in membrane vesicles found within ascites. Furthermore, there are a greater number of these vesicles with increasing disease stage [90]. The utility of exosomes as a minimally invasive biopsy was demonstrated by Runz and colleagues (2007). They determined whether CD24, which is a currently established marker for ovarian cancer prognosis, is present within exosomes. It was revealed that *in vitro* tumor cell-derived exosomes contained CD24 and that it was also present in exosomes obtained from malignant ascites [91]. Moreover, the miRNA profile of exosomes obtained from patients when compared to tumor tissue profiles from the same patient showed the presence of common miRNA hence highlighting exosomal miRNA as a potential biomarker [47].

### Exosomes and drug resistance

Exosomal research also provides a warrantable avenue in patients that do not respond to standard therapies or patients that present with tumor resistance and/or relapse. Recurrence often occurs between 12-24 months of treatment in advanced disease patients and is accompanied by chemotherapy resistant disease [92]. Research by Au Yeung et al (2016) showed that exosomal miRNA (specifically, miR-21) could induce chemo-resistance in ovarian cancer cells. Thus, they suggested that preventing the transfer of miR-21 by exosomes could suppress tumor growth. Literature has also shown that exosomes may have a part in drug resistance as they can increase fibroblast growth which causes a desmoplastic reaction leading to a disruption in treatment delivery [93]. Furthermore, exosomes may also provide a pathway to actively remove drugs leading to a pro-tumorigenic environment.

Safaei et al showed that a cisplatin resistant ovarian cancer cell line released exosomes which had an increased expression of LAMP1 compared to a cisplatin sensitive cell line [94]. LAMP1 is lysosome-associated protein 1 which is a marker associated with lysosomal vesicles (site for cisplatin localisation). Therefore, it was suggested that exosomes were trafficking proteins important for lysosome function thus influencing the development of cisplatin resistance [94]. Yin and colleagues (2012) also proposed a role for exosomes in aiding platinum resistance in ovarian cancer cells [95]. They reported that over-expression of Annexin 3 (A3) provided a pathway for the development of platinum resistance. Fundamentally, they showed that cisplatin resistant ovarian cancer cells released higher amounts of exosomes and that these exosomes contained A3. They also proposed that A3 had a role in the production and fusion of multivesicular bodies leading to the release of exosomes. In contrast to the role of exosomes in drug resistance, exosomes have also been exploited as endogenous nano-carriers for targeted treatments.

### Exosomes as drug delivery vehicles

In addition to their applications in diagnosis and treatment, exosomes provide an invaluable tool for drug delivery and monitoring response. This is attributed to the fact that exosomes can encapsulate and transport molecules through compartmentalisation [93]. Specific drug delivery vehicles are appealing as ovarian cancer treatments are often non-specific leading to several side effects. For therapeutic purposes, exosomes can be produced in large quantities using cells with high proliferative capabilities e.g. mesenchymal stem cells (MSC) [96]. These exosomes present a natural drug delivery vehicle as they do not have adverse effects and may be able to target specific tissues [97]. The advantages of using MSC derived exosomes as drug carriers have been discussed in depth by Yeo et al (2013) [96]. Exosomes are also ideal modes of drug delivery as they have a long circulating half-life and biocompatibility [97]. Furthermore, exosomes can surpass the blood brain barrier.

Research by Yang et al (2015) incorporated anti-cancer drugs, doxorubicin, and paclitaxel, into exosomes and then introduced the exosomes into zebrafish embryos [98]. As a control, they treated the embryos with the drugs alone and found that the drugs were not able to penetrate the blood brain barrier. However, significant amounts of exosomes could penetrate the blood brain barrier leading to cellular uptake of the drugs. Although they were successful in encapsulating drugs into exosomes and delivering them to the target site, they suggested issues that need to be overcome. These issues involve: standardised procedures for isolation and characterisation of exosomes, loading the drugs into exosomes, using peptides to reach the target cells and exosome production from parent cells [98]. Current methods for incorporating substances into exosomes include incubation, electroporation, chemical reagents, and transfection [64]. These methods are discussed in detail in Johnsen et al (2014).

Another study by Tian and colleagues (2014) delivered exosome encapsulated doxorubicin to solid tumors in a mice model [99]. They targeted the exosomes to the tumor cells using an iRGD peptide on the exosomal surface which binds specifically to αv integrins on tumor cells. Incredibly, it was shown that there was a decrease in tumor growth when exosome encapsulated doxorubicin was used compared to an equivalent dosage of free doxorubicin [99]. Exosomes have shown great potential in aiding the premise of personalised medicine as they are equipped with several advantages including an ability to evade the immune system and to carry nano-molecules. It is hoped that the utilisation of exosomes to provide targeted drug delivery remains an active area of research as exosomes represent a non-toxic drug delivery vehicle.

### Using exosomes as tools for real-time monitoring of response to therapy

It has been suggested that circulating exosome levels before and after chemotherapy can be considered to determine a patient’s response to the treatment. Szajnik et al (2013) showed that patients that did not respond to treatment with carboplatin and paclitaxel showed no significant change in exosomal protein levels after treatment compared to prior to treatment [100]. Conversely, patients that had a response to the treatments showed either an increase or decrease in exosomal protein levels [100]. Tumor-derived exosomes are also being examined as although exosomes are released under normal physiological conditions, they are found in greater numbers in patients with disease. A possible explanation for this increase is the idea that stressed tumor cells under hypoxia may be releasing signals via exosomes [70]. Since exosomal cargo can have both an immuno-inhibitory and immuno-stimulatory effect, exosomes provide a less invasive method of analysing the immune response to both the tumor and to potential treatments [70].

Whiteside (2015) has suggested that the immuno-suppressive molecular profile of tumor exosomes could provide an understanding of the immune dysfunction resulting from the tumor and ultimately tumor progression [70]. This was based on data which showed that the immune-competence of cancer patients can be used to predict outcomes [101, 102]. Overall, exosome concentration and exosomal cargo have been proposed as prognostic biomarkers to monitor the response to treatments. Being aware of treatment response is beneficial as it allows the design of treatment plans suited to the patient rather than general treatment plans. Furthermore, using exosomes in this context may also allow differentiation between patients that will respond to a particular treatment and patients that may not respond.

## CONCLUDING REMARKS

Most significantly, it has been proposed in literature that tumor-derived exosomes may provide a way forward in aiding early diagnosis, drug delivery and monitoring response to treatment in the field of ovarian cancer. Furthermore, exosomal cargo including proteins and miRNA may also provide an insight into the cellular environment including changes in the cell at a molecular level. This ability to gain a snapshot of the internal environment in a timely manner may eradicate the need for invasive biopsies and allow detection of disease prior to metastatic spread. Therefore, exosomes provide a minimally invasive “liquid biopsy” method to gain insight into the tumor microenvironment without the need for a highly invasive tumor biopsy.

**Figure 1:**
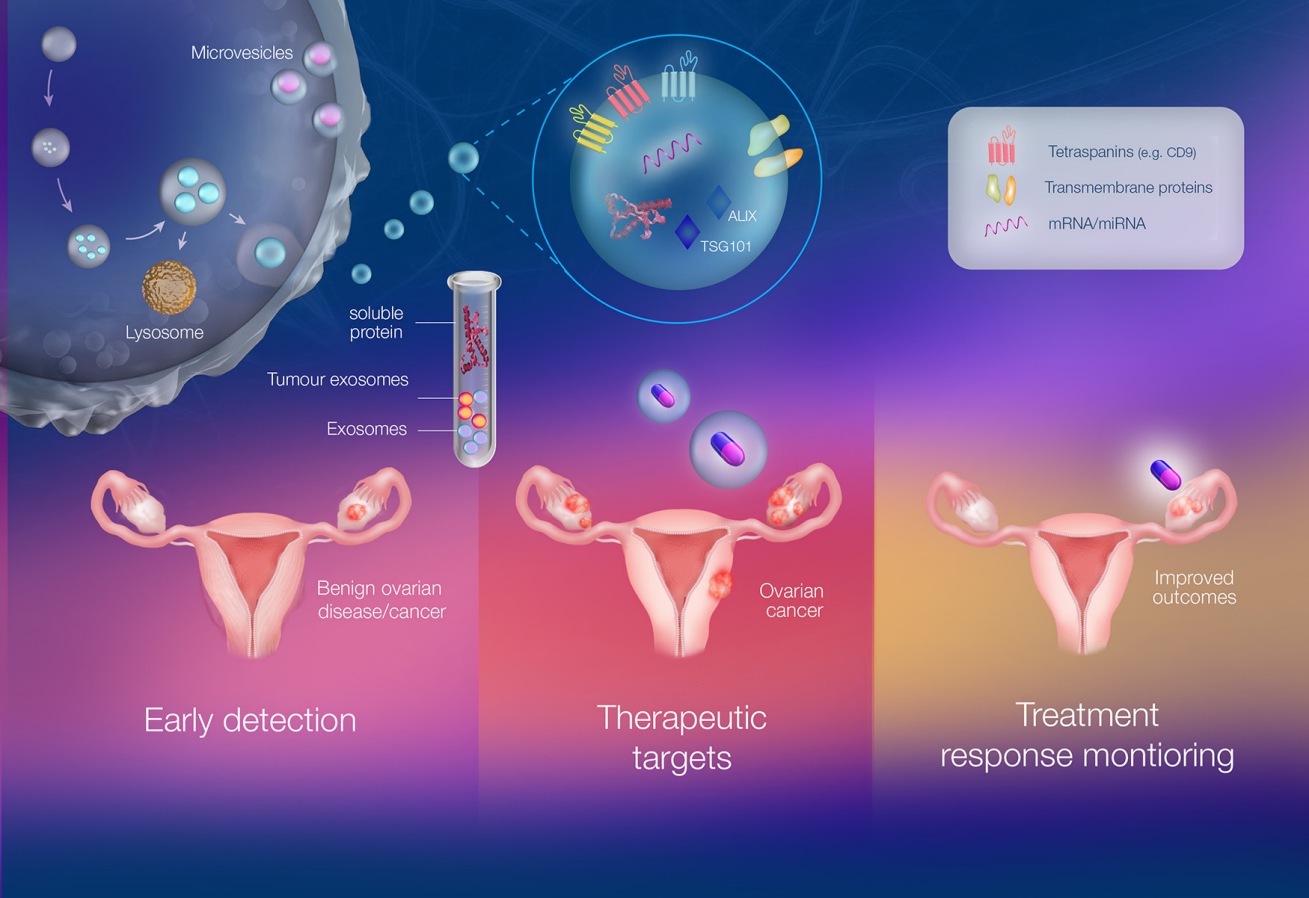
The multi-faceted role of exosomes as liquid biopsies in ovarian cancer involving detection, treatment and monitoring Exosomes are membranous extracellular vesicles of an endocytic origin. They have been implicated in both physiological and pathophysiological conditions. Exosomes are highly stable and are involved in cell-cell communication and can be considered “fingerprints” of the releasing cell. They also have a capacity to evade the immune system and thus do not elicit an immune response. Exosomes can be found in circulation as well as several bodily fluids such as plasma, urine and saliva allowing for easy identification. The circulating exosomes, once separated from abundant proteins and other vesicles, can be examined under different contexts such as early detection, therapeutics and monitoring cellular response to drugs. This is significant in ovarian cancer as there is currently a lack of early detection methods which is a key contributor to the high mortality rates. Current detection tools include measuring CA-125 levels and Transvaginal ultrasounds. Therefore, exosome concentration, exosomal proteins and miRNA (e.g. exosomal CA-125, EpCAM+ exosomes) have been proposed as early detection tools. Exosomes also provide an avenue for personalised medicine as they are often termed “tumor signatures” and can thus be used as minimally invasive biopsies or natural drug delivery vehicles. Finally, circulating exosome levels and/or exosomal protein profile after chemotherapy/ treatment can also be used to monitor response to treatment. Thus, exosomes are emerging as liquid biopsies in the context of ovarian cancer to improve survival rates and patient outcomes.

However, this achievement is dependent on a complete understanding of exosome biogenesis, secretion, interaction with target cells and the pathways underlying exosomal cell signalling. Additionally, it is important to note that exosomal cargo varies, depending on the releasing cell, and therefore, the process of cargo packaging into exosomes also needs to be fully elucidated. In terms of ovarian cancer, the cross-talk between cells and the microenvironment as well as the involvement of exosomes in facilitating this cross-talk also needs to be studied. This review provided a glimpse at the multi-faceted nature of exosomes by discussing recent publications which have aimed to decode the role of exosomes by examining the exosomal cargo, specifically miRNA and proteins. It is anticipated that this review will highlight exosomes as a precious biomedical tool under various contexts as shown in Figure 1 and that research will focus on gaining a deeper understanding of exosomes, exosomal content and their connection with the tumor cells and microenvironment.
